# A New Dicynodont (Therapsida: Anomodontia) from the Permian of Southern Brazil and Its Implications for Bidentalian Origins

**DOI:** 10.1371/journal.pone.0155000

**Published:** 2016-05-25

**Authors:** Alessandra D. S. Boos, Christian F. Kammerer, Cesar L. Schultz, Marina B. Soares, Ana L. R. Ilha

**Affiliations:** 1 Instituto de Geociências, Universidade Federal do Rio Grande do Sul, Porto Alegre, Rio Grande do Sul, Brazil; 2 Departamento de Ciências Naturais, Fundação Universidade Regional de Blumenau, Blumenau, Santa Catarina, Brazil; 3 Museum für Naturkunde, Leibniz-Institut für Evolutions- und Biodiversitätsforschung, Berlin, Germany; Institute of Botany, CHINA

## Abstract

Dicynodonts were a highly successful group of herbivorous therapsids that inhabited terrestrial ecosystems from the Middle Permian through the end of the Triassic periods. Permian dicynodonts are extremely abundant in African deposits, but are comparatively poorly known from the other regions of Gondwana. Here we describe a new South American dicynodont, *Rastodon procurvidens* gen. et sp. nov., from the Boqueirão farm site of the Rio do Rasto Formation, Paraná Basin, Guadalupian/Lopingian of Brazil. Diagnostic features of *R*. *procurvidens* include uniquely anteriorly-curved maxillary tusks, well-developed ridges extending from the crista oesophagea anteriorly along the pterygoid rami, strong posterior angulation of the posterior pterygoid rami, and a bulbous, well-developed retroarticular process of the articular. Phylogenetic analysis indicates that *R*. *procurvidens* is the earliest and most basal member of Bidentalia, a cosmopolitan clade that includes Permian and Triassic dicynodonts whose dentition is usually reduced to a pair of maxillary tusks.

## Introduction

Anomodont therapsids initially radiated during the middle Permian (Guadalupian), and rapidly became the most abundant group of herbivorous terrestrial vertebrates. The anomodont subclade Dicynodontia was particularly successful, with a worldwide distribution and lengthy stratigraphic range extending into the Upper Triassic [1–4]. The Beaufort Group of South Africa bears the richest strata in terms of the abundance and diversity of dicynodonts [5], but they are also known from the Permian deposits of Brazil, China, India, Laos, Madagascar, Malawi, Mozambique, Russia, Scotland, Tanzania, Zambia and Zimbabwe [1,5–18]. Triassic occurrences of the group are additionally known from Antarctica, Argentina, Australia, Brazil, China, Germany, India, Madagascar, Mongolia, Morocco, Namibia, Poland, Russia, Tanzania, the United States, and Zambia (Fröbisch [5] and references cited therein, updated with [19–24]). Because of their abundance, wide geographical distribution and relatively short species-level temporal ranges (with some notable exceptions, e.g., *Diictodon feliceps*), dicynodonts have been extensively used in vertebrate biostratigraphy, especially for intrabasinal and transcontinental correlations (e.g., [25, 26]).

In Brazil, dicynodonts have only been reported from the Paraná Basin in the southeastern part of the country. Permian records consist of only one specimen from the Rio do Rasto Formation (Guadalupian/Lopingian) assigned to *Endothiodon* [27]. By contrast, Triassic dicynodonts are represented by many specimens in three genera: *Dinodontosaurus*, *Stahleckeria* and *Jachaleria* from the Santa Maria Supersequence (Middle/Upper Triassic) [28, 29]. No Brazilian-endemic dicynodont genera are currently known, with *Endothiodon* being particularly widespread (it is also known in India [30], southern and southeastern Africa; Cox and Angielczyk [17]), *Dinodontosaurus* and *Jachaleria* occurring in Brazil and Argentina [28], and *Stahleckeria* occurring in Brazil and Namibia [23].

The specimen here described was previously figured by Dias-da-Silva (Fig 4 in [31]) and mentioned by Boos et al. [27,32] but has never received a formal description. It represents the second dicynodont taxon from the Permian of South America and by far is the best preserved specimen, comprising an almost complete skull with lower jaws and much of the postcranium.

## Geological Setting

**Fig 1:**
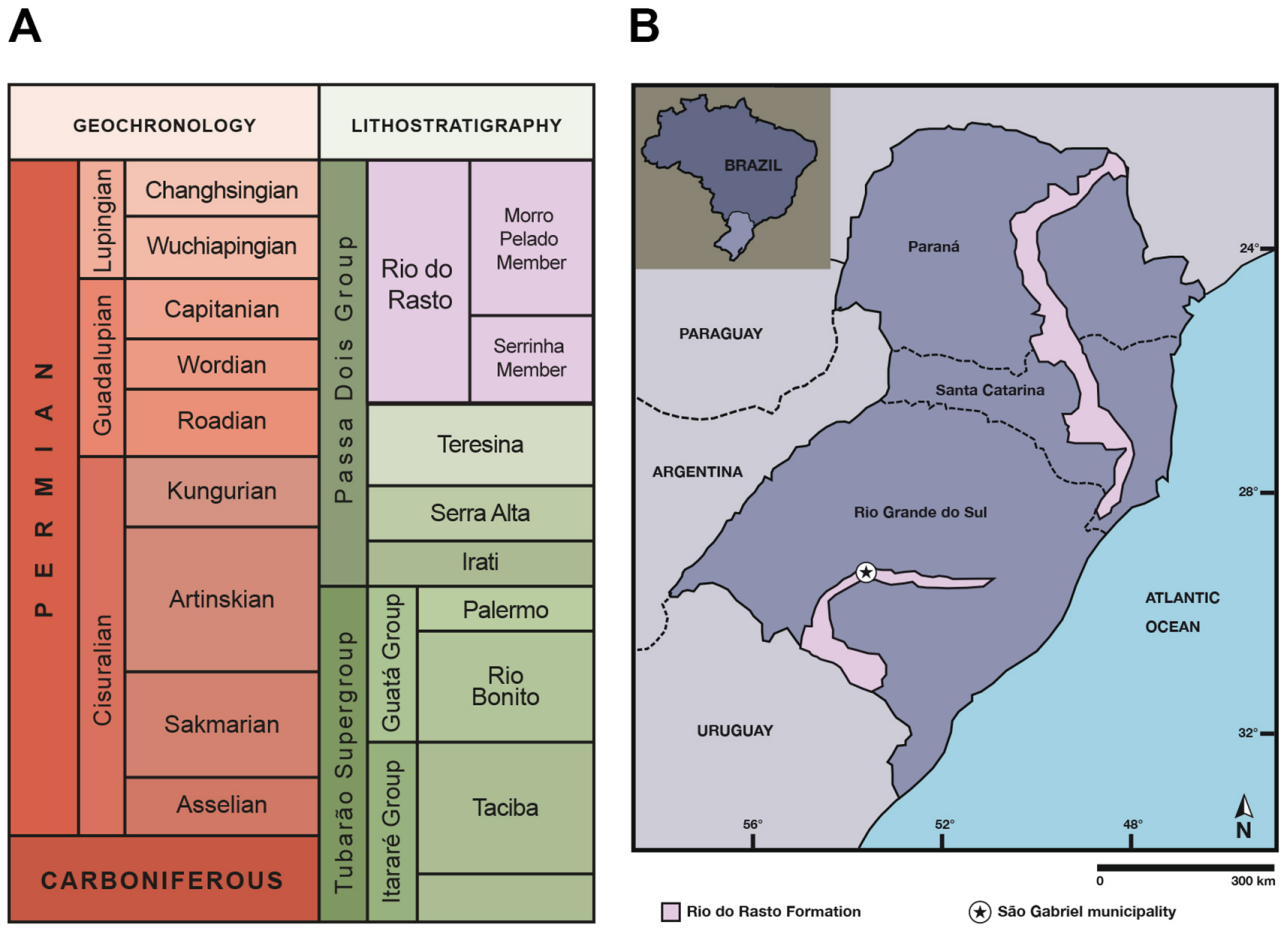
Geological context of the study area. (A) Stratigraphic context of the Permian units of the Paraná Basin in Brazil (based on [35]); (B) Location map of the Rio do Rasto Formation in southern Brazil, indicating the municipality of São Gabriel where the study area (Boqueirão farm) is located. This drawing is similar to [40] but not identical to the original source, and is therefore for illustrative purposes only.

The Paraná Basin is an intracratonic basin (approximate area of 1,500.000 km^2^) that extends over parts of Brazil, Argentina, Paraguay and Uruguay [33]. In Brazil, it is made up of deposits from Ordovician to Cretaceous age, divided into six supersequences (from base to top): Rio Ivaí (Ordovician-Silurian), Paraná (Devonian), Gondwana I (Carboniferous-Early Triassic), Gondwana II (Middle to Late Triassic), Gondwana III (Late Jurassic-Early Cretaceous) and Bauru (Late Cretaceous) [34]. The Permian interval is recorded in rocks belonging to the Gondwana I Supersequence, made up of the top of the Itararé Group and the Guatá and Passa Dois groups [33] (Fig 1A). In the upper portion of the Passa Dois Group is located the Rio do Rasto Formation, the first unit bearing terrestrial tetrapods in the Paraná Basin, which is of Guadalupian/Lopingian age [35] (Fig 1A).

The depositional history of the Rio do Rasto Formation is mainly interpreted as continental, comprising lacustrine, fluvial and aeolian deposition [33,36–38]. The formation is divided into two members: Serrinha (lower) and Morro Pelado (upper). The former consists of fine sandstones with coarser grains at the base but fining upwards with the eventual occurrence of mudstone and siltstone [39], whereas the Morro Pelado Member is characterized by fine to medium-grained sandstone with pelitic intercalations [35,39]. On the top of the Morro Pelado Member there is an increase in the deposition of sandstone layers, pointing to a trend of growing aridity [36]. Tetrapod remains have been found in the fluvio-deltaic facies of the Morro Pelado Member in Paraná, Santa Catarina and Rio Grande do Sul states, and include temnospondyl amphibians, parareptiles and therapsid synapsids [40]. Fossil occurrences in the Morro Pelado Member are not restricted to tetrapods, but also include fish remains (e.g. [41–42]), ichnofossils (e.g. [43–44]), plants (e.g. glossopterids, pecopterids, sphenophytes) (e.g. [45]) and invertebrates (e.g. bivalves, conchostracans) (e.g. [46]).

The dicynodont material described here was found at the Boqueirão farm outcrop, located in the municipality of São Gabriel (Catuçaba district), approximately in the central part of the Rio Grande do Sul State (Fig 1B), inside a private property. The fossil was embedded in pinkish fine sandstone and the bones were covered by a dark oxidized crust. A pond separates the two exposures of the Morro Pelado Member of the Rio do Rasto Formation at the site. The SW outcrop contains only coprolites, whereas the NE outcrop yields tetrapod remains [Paula Dentzien-Dias, pers. com. 2016], such as the dicynodont described here, the dinocephalian *Pampaphoneus biccai* and a temnospondyl amphibian [31, 47, 48]. The layers of the NE outcrop are tilted (with a SW dip), whereas those of the SW outcrop are horizontal, so it is not clear if these two exposures are coeval.

The presence of *Pampaphoneus* points to a Guadalupian age for the NE outcrop based on its affinities with anteosaurid dinocephalians of South Africa and the Russian Platform [48]. The phylogenetic position of the new dicynodont here described is also concordant with such an age.

## Methods

### Ethics statement

All necessary permits were obtained for the described study, which complied with all relevant regulations. Permission to excavate the specimens from the Boqueirão farm site was obtained from the landholders by the team from the Universidade Federal do Pampa (UNIPAMPA) of São Gabriel municipality in 2010. The specimen UNIPAMPA PV317P is currently housed in the collections of the Laboratório de Paleobiologia of UNIPAMPA, in São Gabriel, Rio Grande do Sul State, Brazil. Permission to access and study the specimen here described was granted to the co-authors by the curator of the aforementioned collection, Dr. Felipe Lima Pinheiro.

### Nomenclatural acts

The electronic edition of this article conforms to the requirements of the amended International Code of Zoological Nomenclature, and hence the new names contained herein are available under that Code from the electronic edition of this article. This published work and the nomenclatural acts it contains have been registered in ZooBank, the online registration system for the ICZN. The ZooBank LSIDs (Life Science Identifiers) can be resolved and the associated information viewed through any standard web browser by appending the LSID to the prefix “http://zoobank.org/”. The LSID for this publication is: urn:lsid:zoobank.org:pub:60995DB7-406B-458C-87C1-ECFC49FA6816. The electronic edition of this work was published in a journal with an ISSN, and has been archived and is available from the following digital repositories: PubMed Central (http://www.ncbi.nlm.nih.gov/pmc/) and LOCKSS (http://www.lockss.org).

### Preparation

The specimen was prepared using both chemical and mechanical methods. For mechanical preparation, matrix was removed using micro pneumatic hammers whereas iron oxide layers were removed with the help of a small metal chisel. Chemical preparation was done by baths in hydrogen peroxide solution.

### Phylogeny

We included UNIPAMPA PV317P in the most recent version (Kammerer et al. [49]) of the anomodont data matrix of Kammerer et al. [4], composed of 174 characters and 102 taxa. However, the specimen studied herein was only coded for cranial and mandibular characters; postcranial characters will be considered in the future, upon the full description of the *Rastodon* postcranium. The analysis was run in TNT v1.1 [50] using New Technology search parameters (sectorial searching, parsinomy ratchet, tree drift, and tree fusing utilized; search level set to 65; required to find minimal tree length 20 times). Symmetric resampling values are based on 10000 replicates in TNT.

## Systematic Paleontology

Therapsida Broom, 1905 [51]

Anomodontia Owen, 1860 [52]

Chainosauria Nopcsa, 1923 [53]

Dicynodontia Owen, 1860 [52]

Bidentalia Bain *vide* Owen, 1876 [54]

*Rastodon* gen. nov.

urn:lsid:zoobank.org:act:2846B384-9D10-4EFD-99F0-C1A7FBB5AE55

### Etymology

A combination of ‘Rasto’ (from Rio do Rasto Formation) and ‘odon’ (from ancient Greek, tooth).

### Diagnosis

As for type and only species.

*Rastodon procurvidens* sp. nov.

urn:lsid:zoobank.org:act:D35AFEAF-1FB6-4705-A024-7CA935C8716F

### Etymology

‘Procurvidens’ from the Latin procurvus (curved forward) and dens (tooth), in reference to the unique anteriorly-curved tusks of this taxon.

### Holotype

UNIPAMPA PV317P, an almost complete skull with attached lower jaws and unprepared postcranium.

### Locality and horizon

Boqueirão farm (S 30 0 6.394; W 54 5 10.067), São Gabriel municipality, Rio Grande do Sul State. Exposure of the Morro Pelado Member (Guadalupian/Lopingian) of the Rio do Rasto Formation, Paraná Basin. Other tetrapods known from the Boqueirão farm locality are the anteosaurid dinocephalian *Pampaphoneus biccai* [48] and the temnospondyl amphibian *Konzhukovia sangabrielensis* [47].

### Diagnosis

Small dicynodont characterized by autapomorphic maxillary caniniform morphology (embayment in ventrolateral margin of caniniform process surrounding small tusks that are strongly curved such that their tips point anteriorly) and the following unique combination of characters: well-developed ridges extending from the crista oesophagea anteriorly along the pterygoid rami, strong posterior angulation of the posterior pterygoid rami, snout with median nasal boss confluent with premaxilla, median ridge present on premaxilla, squamosal folded anteriorly, dorsal to quadratojugal, postorbitals extending to posterior end of the temporal fenestra that remain broad at tip, collar-like boss surrounding the pineal foramen, elongate intertemporal bar with parietals broadly exposed in median groove, narrow, slit-like mandibular fenestra, thin lateral dentary shelf forming the dorsal border of the mandibular fenestra, and well-developed, bulbous retroarticular process of the articular.

## Description

The holotype and only specimen of *Rastodon procurvidens* (UNIPAMPA PV317P) is a somewhat dorsoventrally flattened skull of small size (basal skull length: 86 mm) with the lower jaw rami occluded with the palatal surface of the skull. The specimen also includes a series of unprepared postcranial elements: cervical vertebrae, ribs, clavicle, interclavicle, humerus, radius, ulna, phalanges, pelvic girdle, femur, and tibia. The postcranium will be described in a future contribution.

### Skull in dorsal and lateral views

**Fig 2:**
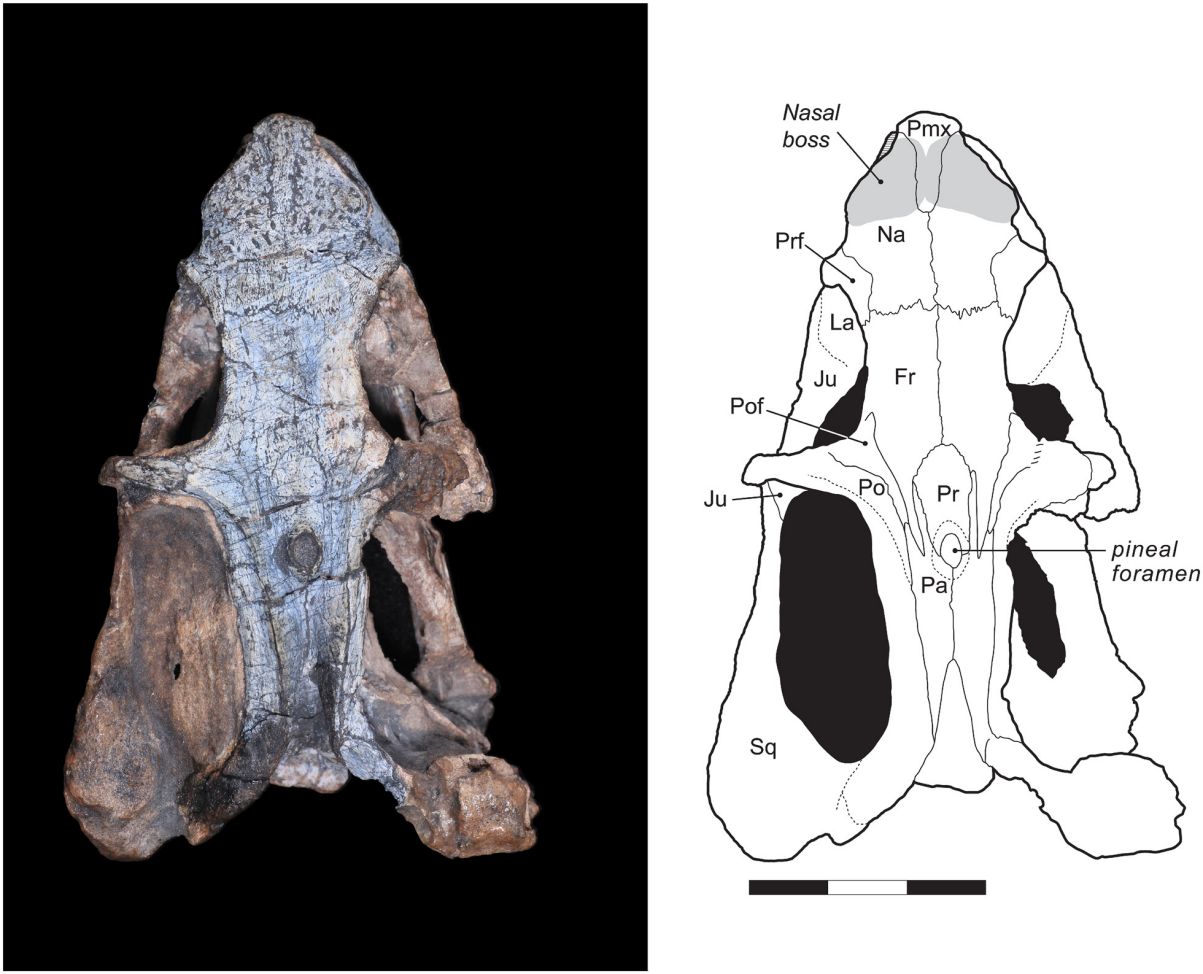
*Rastodon procurvidens* in dorsal view. Photograph (left) and interpretative drawing (right). **Fr**, frontal; **Ju**, jugal; **La**, lacrimal; **Na**, nasal; **Pa**, parietal; **Pmx**, premaxilla; **Po**, postorbital; **Pof**, postfrontal; **Pr**, preparietal; **Prf**, prefrontal; **Sq**, squamosal. Scale bar equals 3 cm.

The overall skull morphology of UNIPAMPA PV317P is typical for a dicynodont, with a shortened pre-orbital region, zygomatic arch emarginated upwards, lateral process of the pterygoid directed forwards [2], and large temporal fenestra. This specimen is somewhat dorso-ventrally compressed, resulting in some distortion of the orbits (more ovoid in lateral view and exposed dorsally than would be the case in the living animal) and the tip of the snout, which is compressed against the anterior portion of the dentaries (Fig 2).

**Fig 3:**
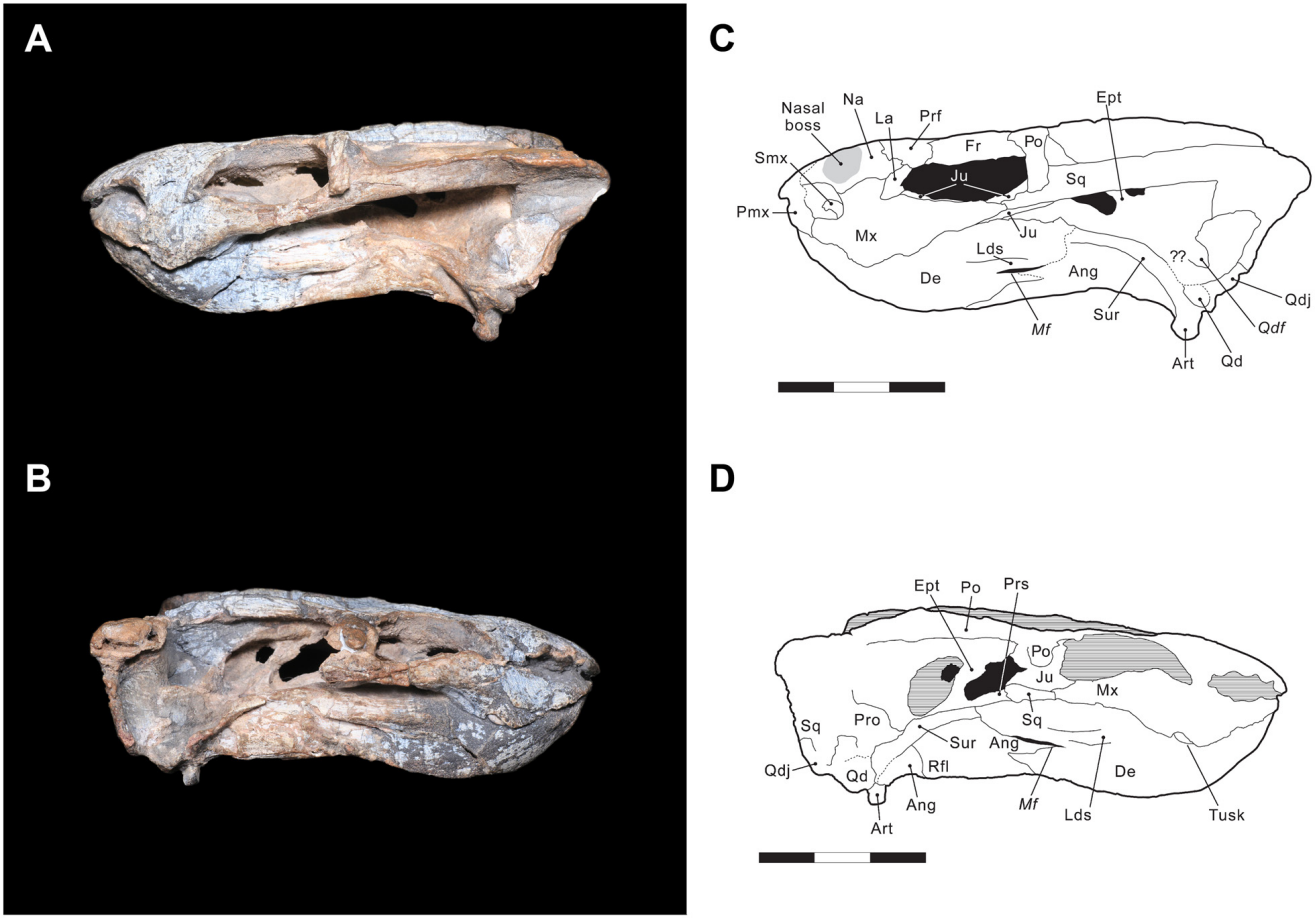
*Rastodon procurvidens* in lateral views. Photographs (A and B) and interpretative drawings (C and D). **Ang**, angular; **Art**, articular; **De**, dentary; **Ept**, epipterygoid; **Fr**, frontal; **Ju**, jugal; **La**, lacrimal; **Lds**; lateral dentary shelf; ***Mf***, mandibular fenestra; **Mx**, maxilla; **Na**, nasal; **Pmx**, premaxilla; **Po**, postorbital; **Prf**, prefrontal; **Pro**, prootic; **Prs**, presphenoid; **Qd**, quadrate; ***Qdf***, quadrate foramen; **Qdj**, quadratojugal; **Rfl**, reflected lamina of angular; **Smx**, septomaxilla; **Sq**, squamosal; **Sur**, surangular. Scale bars equal 3 cm.

Anteriorly, the tip of the snout consists of the premaxillae, which are completely fused into a single element. A smooth median ridge is visible on the anterodorsal face of the premaxillae. A similar structure is also known in the emydopid *Emydops oweni* [55] and pylaecephalid *Prosictodon dubei* [56]. The dorsal process of the premaxilla is narrow and elongate, and separates the nasals for nearly half of their length. Due to breakage, the anteriormost portion of the premaxilla is missing. The anterior margin of the external nares is bordered by the premaxilla, whereas the posterior margin is made up of the nasal and the maxilla. Within the narial opening is located the septomaxilla, which has a semicircular shape and is only preserved on the left side of the specimen (Fig 3A and 3C).

Laterally, the premaxilla contributes to the anterior margin of the naris and has a small contact with the septomaxilla. Slightly posterior to this contact, the premaxilla contacts a small portion of the maxilla. There appear to be no premaxillary teeth—although the ventral surface of the premaxilla is obscured by the dentary, premaxillary teeth are absent in nearly all dicynodonts.

On the dorsal surface of the skull, the nasals are bordered posteriorly by the frontals and posterolaterally by the prefrontals. The contact with the frontals is marked by an interdigitated transverse suture. Each nasal bears a boss, bulging laterally, and these bosses are united medially with the premaxilla to form a single raised region. Nasal bosses are broadly distributed across Dicynodontia, and median nasal bosses are common in basal dicynodonts such as *Pristerodon* and pylaecephalids [4,57–60]. Boss size in dicynodonts seems to be correlated with the maturity of the individual (with larger specimens bearing better-developed bosses), and also with sexual dimorphism (see Tollman et al. [61] for a discussion on *Aulacephalodon* and Kammerer et al. [62] for *Pelanomodon*). Lateral contact between the nasal and the maxilla and lacrimal are best preserved on the left side of the specimen (Fig 3A and 3C). The surface of both premaxilla and nasals is ornamented, bearing a dense array of foramina, which are thought to be associated with a keratinous covering of these structures in life [63].

The prefrontal is exposed dorsally and laterally on the skull and contributes to the anterodorsal margin of the orbit. The prefrontal contacts the nasal and lacrimal ventrally and the frontal medially and posteriorly (Fig 3C). A marked notch in the lateral outline of the snout is observed between the nasals and the prefrontals in dorsal view (Fig 2).

The maxilla is the largest element of the lateral side of the snout. It contacts the premaxilla and septomaxilla anteriorly, and the nasal and lacrimal dorsally. Posteriorly, the maxilla extends onto the zygomatic arch, contacting the jugal, but not contributing to the ventral rim of the orbit. It terminates immediately posterior to the midpoint of the orbit. A maxillary contact with the zygomatic process of the squamosal is preserved on both sides of the skull.

**Fig 4:**
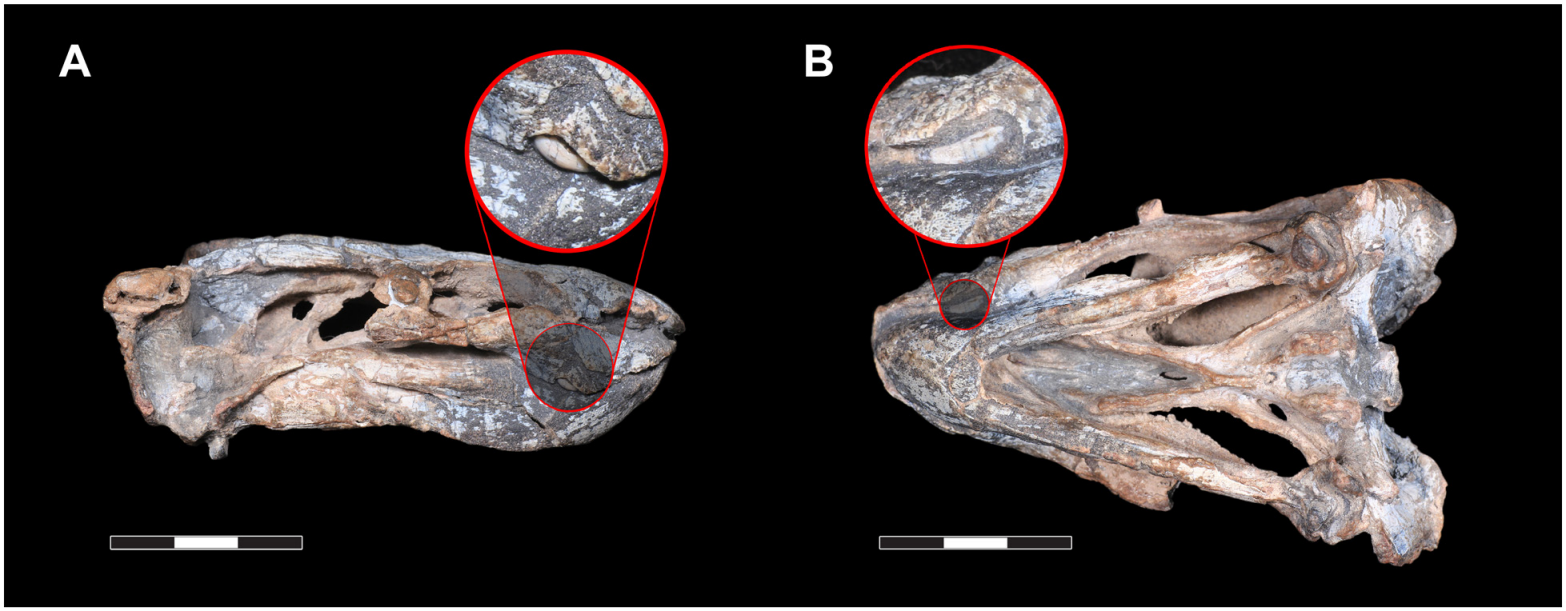
*Rastodon procurvidens* tusks. Photographs of the specimen in right lateral (A) and ventral (B, left side up) views, with a close-up of the caniniform tusks showing their curved morphology and tips directed anteriorly. Scale bars equal 3 cm.

The maxilla bears a ventrally-directed caniniform process, as is typical for dicynodonts. Unusually, however, a distinct ventro-lateral embayment is present on the underside of the caniniform process, housing the tusk. The tusk is extremely small and, uniquely among dicynodonts, strongly curved forwards (procurved), with the tip being directed anteriorly (Fig 4A). This morphology does not appear to be pathological, as the same tusk shape is present on both sides of the skull and the curved tusk fits perfectly into the caniniform embayment. This tusk shape cannot be explained by taphonomic deformation, either. The tusks are gradually curved, and show very faint longitudinal striations that match the curvature of the tusk as a whole. If their shape was the result of deformation, we would expect them to be cracked, as teeth are resistant to plastic deformation, much more so than surrounding bone. Even in the enormous sample of *Diictodon* skulls from South Africa, dorsoventrally compressed specimens rarely show deformation of the tusks themselves, and if so cracking is always evident (C. Kammerer pers. obs.). The function of these procurved tusks is unclear, although based on their position they must have contributed to the masticatory stroke of the jaws, as their lingual face would have contacted the lower jaw during propalinal movement (Fig 4B). Procurved teeth of unknown function are known in another dicynodont, the enigmatic Tanzanian taxon *Abajudon*, although in that taxon they are postcanines, not tusks [16].

UNIPAMPA PV317P is a small specimen, and its tusk bases are smaller than their surrounding alveoli (Fig 4B), so they may be newly erupting. No major changes in tusk angulation between initial eruption and maturity are known in other dicynodonts, however, so this is unlikely to account for their unusual curvature in *Rastodon*. While tusk presence/absence and relative size vary in a number of dicynodont taxa (either due to dimorphism, as observed in *Diictodon* [64], or intraspecific variation, as observed in *Tropidostoma* [65]), general tusk shape in those taxa is consistent within species.

Posteriorly, the border of the caniniform process is gently curved upwards. A distinct postcaniniform crest or keel is absent. Posterior to the caniniform process, the palatal rim bears a vascular foramen on each side of the maxilla (*Fo* in Fig 4). On the right maxilla, an additional foramen is located anterior to the aforementioned one. No labial fossae are present. The presence or absence of postcanine teeth cannot be confirmed, because the mandibles are still tightly in place.

The lacrimal is preserved on the left side of the skull, forming the anteriormost portion of the margin of the orbit. The lacrimal margin of the orbit bulges out laterally as a small protuberance; it does not form a massive boss as in geikiids and some lystrosaurids. The lacrimal foramen is visible in this protuberance, at the inner surface of the orbit rim. It does not exit facially. Dorsally, the lacrimal contacts the prefrontal, anteriorly the nasal and the maxilla, and posterolaterally the jugal.

The jugal forms the ventral margin of the orbit and medially and ventrally contributes to the zygomatic arch. The jugal is best preserved on the left side of the specimen (Fig 3A and 3C), where it meets the maxilla and the lacrimal anteriorly and the squamosal posteriorly. On the left lateral surface of the zygomatic arch, between the orbit and the temporal fenestra, a small portion of the postorbital overlies the jugal. Ventrally, contacts with the maxilla and the squamosal are clearly present, but position of the contacts with the palatine and the ectopterygoid are uncertain due to coverage by the mandible.

The frontals are rectangular elements located on the skull roof, extending from the interorbital region to the anterior portion of the intertemporal region. Laterally, the frontal makes up the majority of the dorsal margin of the orbit. The frontals also contact the nasals anteriorly, prefrontals anterolaterally, postfrontals posterolaterally, and the parietals and preparietal posteriorly, but do not closely approach the premaxilla. The naso-frontal suture is nearly straight and weakly interdigitated. The frontal envelops the preparietal posteriorly with an arc-shaped suture. Attenuate posterior process of the frontals extend between the postfrontals and preparietal, terminating between the anterior processes of the parietals at a level immediately posterior to the mid-length of the pineal foramen.

On the right side of the skull roof the postfrontal is weathered, so this description is primarily based on the left side. The postfrontal is triangular in outline with an attenuate posterior process extending between the postorbital and frontal. This element contributes to a small portion of the posterodorsal margin of the orbits. Posteriorly, the postfrontal has a very short contact with the lateral anterior process of the parietal.

The postorbital is also better preserved on the left side of UNIPAMPA PV317P. As in all dicynodonts it is divided into a descending process that makes up the postorbital bar and a posterior process bounding the temporal fenestra. The postorbital bar appears to be made up entirely of postorbital, without a dorsally-directed contribution from the jugal. If a dorsal spur of the jugal is present, it must be small and confined to the (unprepared) medial face of the postorbital bar. The postorbital bar is relatively narrow and unornamented. The posterior process of the postorbital makes up almost all of the medial margin of the temporal fenestra, terminating posterior to the occiput at a contact with the squamosal. Medially, the postorbital has an elevated suture with the postorbital. Laterally it curves downwards, forming a weakly convex face at the margin of the temporal fenestra without a distinct break in slope.

The preparietal is a single median element, roughly ovoid in outline but broadest anteriorly. Posteriorly it forms the anterior half of a collar-like boss that encircles the elliptical pineal foramen. The preparietal is flat and flush with the rest of the skull roof and is surrounded by the frontals anteriorly and bordered laterally and posteriorly by the parietals.

The parietals are exposed only in dorsal view, since they are overlapped laterally by the posterior process of the postorbitals. The dorsal surface of the parietals forms a distinct median groove; the parietals are substantially lower at their median suture than at their lateral border with the postorbitals. Each parietal bears two attenuate anterior processes: a relatively short lateral process that extends between the postorbital and frontal and contacts the posterior end of the postfrontal at tip, and a longer medial process that extends between the frontal and preparietal. Ventrally, a descending flange of the parietal contacts the ascending ramus of the epipterygoid. Near this flange, a fossa for muscle attachment is present on the ventral face of the intertemporal bar. Posteriorly, the parietals contact the postparietal. Although the exact position of this suture is uncertain, it is clear that the postparietal does not make a significant contribution to the dorsal skull roof.

The unpaired postparietal (partially homologous with the interparietal in mammals, see Koyabu et al. [66]) forms part of the sloping occiput between the ridges formed by the postorbitals and parietals.

The squamosals of UNIPAMPA PV147P are made up of three rami, as is typical of dicynodonts, but they are not fully preserved on the right side of the specimen. In dorsal view, the contact between the dorsal process of the squamosal and the postorbital is located where the temporal margin begins curving outwards. The lateral process of the squamosal comprises part of the posterior half of the zygomatic arch and delimits the posterolateral margin of the temporal fenestra. Anteriorly, it contacts the maxilla, jugal, and postorbital. The long posteroventral process of the squamosal has an extensive contact with the quadrate and the quadratojugal, and medially, it contacts the paraoccipital process of the opisthotic. The posteroventral process of the squamosal has an anterior fold dorsal to the quadratojugal, similar to the condition in *Daqingshanodon* [4, 67] and *Brachyprosopus* [68].

The quadratojugal is a plate-like element, broader dorsally and narrower ventrally. Its dorsal surface contacts the squamosal, whereas its medial and ventral portion meets the quadrate.

The quadrates are not fully exposed in UNIPAMPA PV317P, as they are articulated with the posterior region of the lower jaws. Dorsally, the quadrate is partially covered by the squamosal and dorsomedially, the quadrate meets the quadratojugal. A quadrate foramen is visible on both sides of the skull.

The epipterygoid (Fig 3B and 3D) is exposed only on the right side of the specimen, but it exhibits the typical morphology found in other dicynodonts. It is made up of a footplate attached to the dorsal surface of the pterygoids and a thin, anterodorsally-ascending process that reaches the skull roof, contacting the ventral surface of the parietals. A low dorsal process is also present at the anterior end of the epipterygoid footplate.

### Ventral view

**Fig 5:**
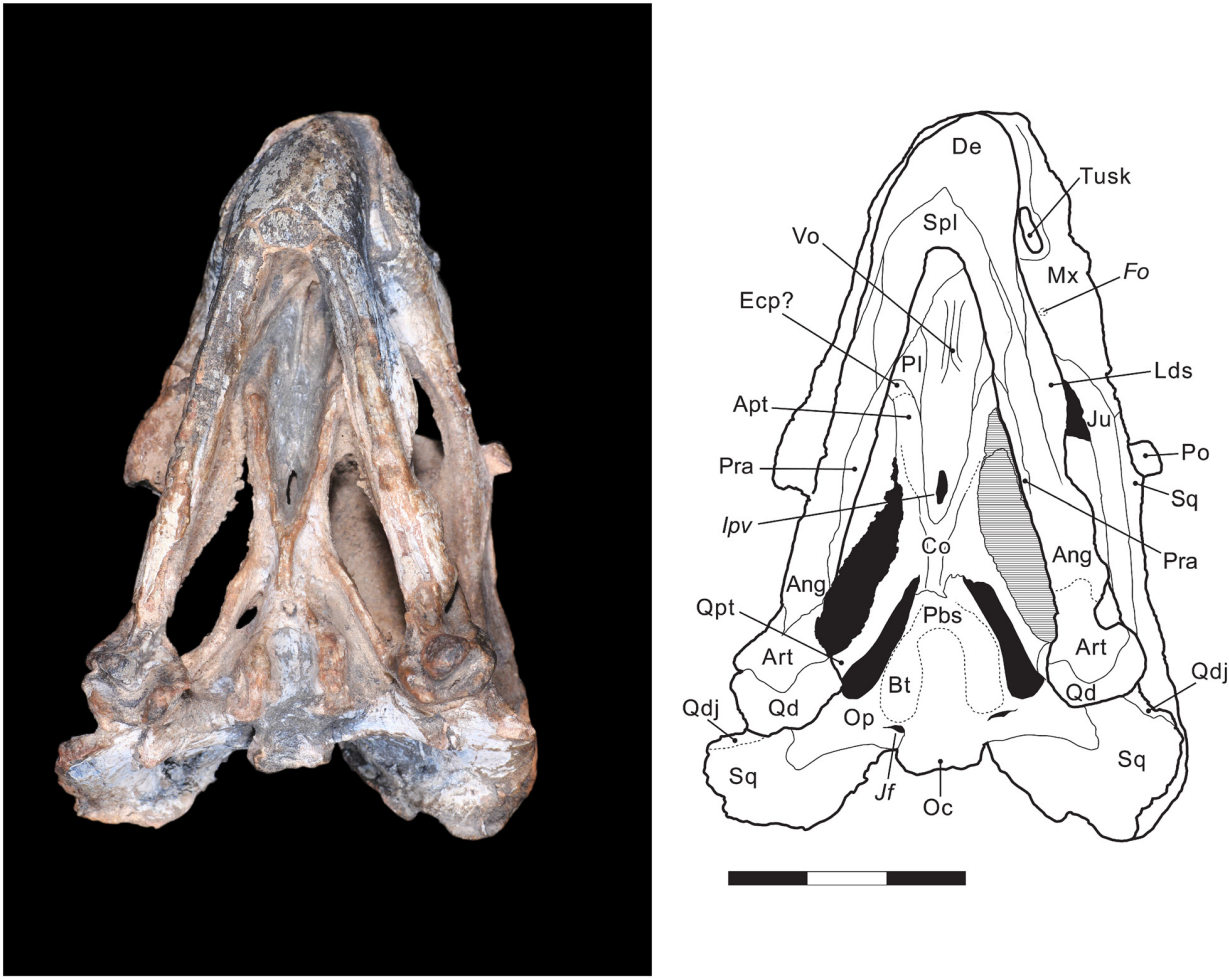
*Rastodon procurvidens* in ventral view. Photograph (left) and interpretative drawing (right). **Ang**, angular; **Apt**, anterior ramus of the pterygoid; **Art**, articular; **Bo**, basioccipital; **Bt**, basal tuber; **Co**, crista oesophagea; **De**, dentary; **Ecp**, ectopterygoid; ***Fo***, foramen; ***Ipv***, interpterygoid vacuity; ***Jf***, jugular foramen; **Ju**, jugal; **Lds**, lateral dentary shelf; **Mx**, maxilla; **Oc**, occipital condyle; **Op**, opisthotic; **Pbs**, parabasisphenoid; **Pl**, palatine; **Po**, postorbital; **Pra**, prearticular; **Qd**, quadrate; **Qdj**, quadratojugal; **Qpt**, quadrate ramus of the pterygoid; **Spl**, splenial; **Sq**, squamosal; **Vo**, vomer. Scale bar equals 3 cm.

Due to the occlusion of the mandible with the skull, the premaxillary and maxillary portions of the palate are not visible. The anteriormost structure visible in ventral view is the vomer. The vomer is visible anterior to the interpterygoid vacuity and has paired ventral ridges that surround a median trough. Little is exposed of the anterior portion of the palatines and the presence of the right ectopterygoid is only tentatively indicated in Fig 5. The exposed posterior portion of the palatine is smooth like in primitive dicynodonts, but it is possible that a more rugose anterior area of the palatine is not visible.

The pterygoids are X-shaped, made up of an anterior (palatal) ramus, median plate and a posterior (quadrate) ramus. A notable feature of *Rastodon* is the strong development of ventral ridges on the anterior rami of the pterygoids. These ridges are present in many dicynodonts, but are unusually tall and discrete in *Rastodon*, converging posteriorly to unite with a very well-developed crista oesophagea on the median plate. These ridges appear to extend far anteriorly, onto the palatines. The anterior ramus of the pterygoid contacts the ectopterygoid and a small portion of the palatine. The median pterygoid plate contributes to the posterior border of the interpterygoid vacuity and is divided medially by the crista oesophagea. The crista oesophagea bifurcates posteriorly, forming two ridges that extend to the anterior margins of the basal tubera. The quadrate ramus arises from the lateral edge of the median plate and posteriorly contacts the medial part of the quadrate. The posterior ramus of the pterygoid is strongly posteriorly-angled in *Rastodon*, contrary to many dicynodonts in which it has a broad lateral splay [4]. On the dorsal surface of the posterior ramus of the pterygoid is located the footplate of the epipterygoid (Fig 3B and 3D).

The parabasisphenoid (an element formed by fusion of the parasphenoid and basisphenoid) is exposed on the ventral surface of UNIPAMPA PV147P. Anteriorly, it meets the median plate of the pterygoid and posteriorly forms part of the basal tubera. There is a shallow, weak anterodorsal slope of the basisphenoid contribution to the tuber towards the median pterygoid plate, which is primitive for dicynodonts [4]. In lateral view (Fig 3B and 3D), the cultriform process of the parasphenoid is visible with a triangular presphenoid attached to its dorsal surface.

Most of the basioccipital bone is exposed on the ventral surface of the specimen, where it makes up roughly half of the elongate basal tubera. No intertuberal ridge is present. The suture between the basioccipital and opisthotic is unclear, and they may be fused to form a periotic element in this taxon.

### Occipital view

**Fig 6:**
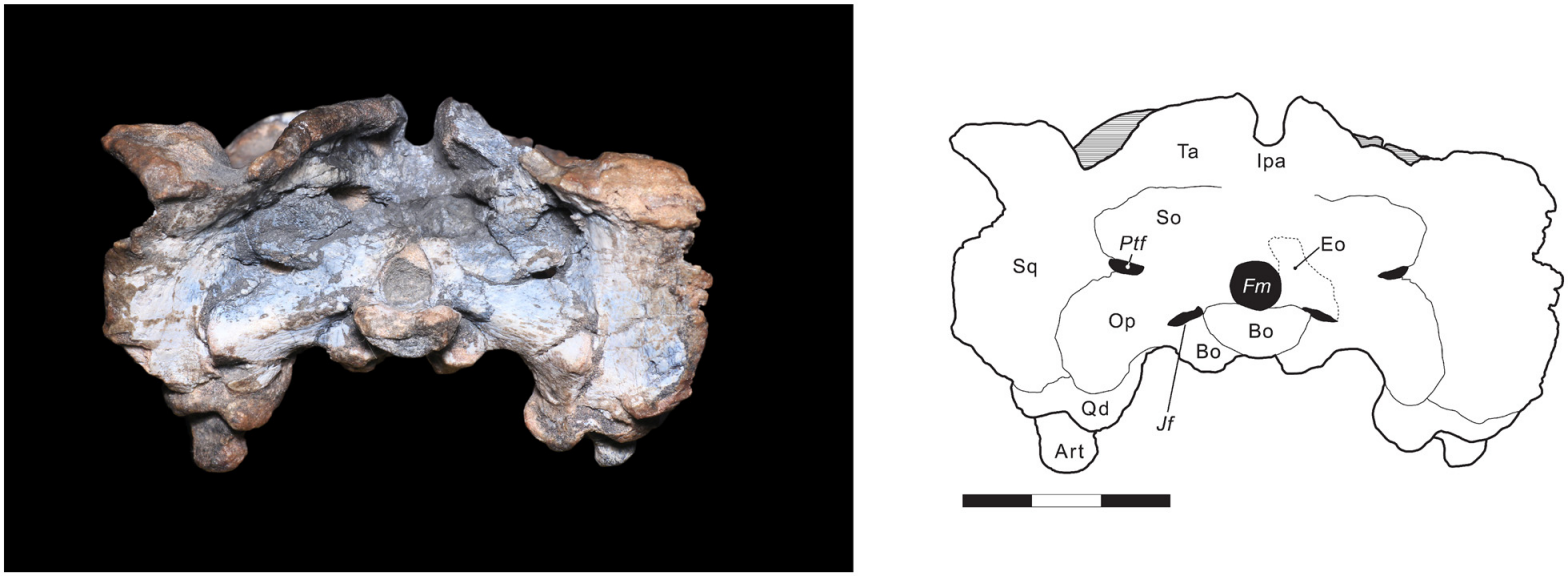
*Rastodon procurvidens* in occipital view. Photograph (left) and interpretative drawing (right). **Art**, articular; **Bo**, basioccipital; **Eo**, exoccipital; ***Fm***, foramen magnum; **Ipa**, interparietal; ***Jf***, jugular foramen; **Op**, opisthotic; **Ptf**, post-temporal fenestra; **Qd**, quadrate; **So**, supraoccipital; **Sq**, squamosal; **Ta**, tabular. Scale bar equals 3 cm.

The occipital plate of UNIPAMPA PV147P is well preserved ventrally, but worn and damaged towards its dorsal margins. The foramen magnum is teardrop-shaped. A post-temporal fenestra is present on both sides of the skull, at the same level as the midpoint of the foramen magnum. It seems that most of the bones of the occipital plate (basioccipital, exoccipital, supraoccipital, opisthotic, and prootic) are fused into a single element, the periotic. Because of this, sutures are difficult to interpret in this region of the skull and the tracings shown in Fig 6 are tentative. The periotic contributes to the dorsal margin of the foramen magnum and to the medial rim of the post-temporal fenestra. A lateral process of the periotic (the portion that would be made up of supraoccipital in an unfused occiput) also contacts the ventral expansion of the squamosal and the ventral border of the tabular. Dorsally, the periotic would meet the ventral margin of the postparietal, however, the shape of this contact is not clear since most of the dorsal border is damaged.

On the dorsal portion of the occipital surface, the postparietal is present, but the bone surface is badly weathered in this area; what is shown in Fig 6 is a tentative interpretation. The tabulars are thin elements exposed on the dorsal portion of the occiput. They are located lateral to the postparietal and medial to the squamosals. Ventromedially, they contact the supraoccipital portion of the periotic.

The occipital condyle is trilobate and presumably composed of the paired exoccipitals that form the two upper lobes and the unpaired basioccipital, which forms the ventral lobe of the condyle. Lateral to the occipital condyle, a jugular foramen is present on each side of the skull where the exoccipital, opisthotic and basioccipital meet.

The paroccipital processes of the periotic (opisthotic in taxa where this element is unfused) extend lateral to the foramen magum. They expand in height laterally before contacting the occipital portion of the squamosal in a broad suture. A weak posterior projection is present in the ventral portion of the paroccipital process near its border with the squamosal. The paroccipital processes notably curved ventrolaterally, such that their tips are located ventral to the occipital condyle and basal tubera. Ventrally, the paroccipital process contacts the posterodorsal edge of the quadrate.

### Mandible

The mandible of UNIPAMPA PV317P consists of two nearly complete rami (Figs 3 and 4). Anteriorly, the two dentaries are fused together across the midline and curved upwards. The anterior surface of the symphysis bears a longitudinal ridge where the two dentaries meet. Most of the dorsal margin of the dentaries is fixed against the palatal region of the skull, making it impossible, at present, to determine whether dentary tables or teeth are present. Posterior to the region covered by the palate, there is a long flattened region on the dorsal surface of the lower jaw. It could represent the posterior dentary sulcus. On the lateral surface of each dentary, a prominent but thin lateral dentary shelf is present. The dorsal surface of the shelf is mostly flat, although there is a depression on the posterodorsal surface of the shelf. Posterior to the shelf, the dentary meets the surangular at the dorsal rim of the jaw and the angular at the ventral rim of the jaw. The mandibular fenestra is elongated and slit-like and bordered posteriorly by the angular and dorsally by the lateral dentary shelf.

The thin and slender splenial is only visible in ventral and medial views. It is fused across its midline, at the posteroventral edge of the jaw symphysis. There, the splenial is taller than its posterior portion, which extends along the medial surface of the dentary rami until it meets the angular and the prearticular. A narrow, triangular anterior process of the splenial extends between the two sides of the dentary at the base of the jaw symphysis (Fig 5).

The ventral margin of the jaws are gently curved where the dentary and the angular meet. The angular bears a reflected lamina, which is best preserved on the right side of the jaw. The reflected lamina is large, considering the proportions of the mandible, and unornamented.

The surangular is visible on both sides of the jaws and forms the dorsal margin of the lower jaw, posterior to the dentary shelf and anterior to the articular. Ventrally, the surangular contacts the angular.

The prearticular is a rod-shaped element most visible on the ventral surface of the lower jaws. Anteriorly, it contacts the angular and the splenial and posteriorly, it probably meets the articular though the suture with the latter is not visible. It seems clear that the prearticular also contributed to the medial surface of the mandible, but the way the specimen is preserved does not allow a good view of the region.

The articular is the posterior-most element of the jaw and it is attached to the skull with the quadrate. It contacts the surangular anterodorsally and probably the prearticular medially. It is very similar in shape with the articular found in most dicynodonts, with two articular surfaces separated by a median ridge in dorsal view. Anteriorly, the articular forms part of a relatively horizontal articular recess with the surangular. Posteriorly, the articular bears a large, rounded, ventrally-directed retroarticular process.

## Discussion

### Comparisons with other basal dicynodonts

Aside from its unique tusk morphology, *Rastodon procurvidens* exhibits a very generalized dicynodont skull morphology. Even beyond the tusks, however, this taxon can clearly be distinguished from all other dicynodonts based on its unique combination of cranial characters. Distinctions between *Rastodon* and highly autapomorphic dicynodont taxa such as endothiodontids and ‘higher’ cryptodonts and dicynodontoids (i.e., geikiids, rhachiocephalids, lystrosaurids, and kannemeyeriiforms) are self-evident (refer to Kammerer and Angielczyk [59], Kammerer et al. [4], and Cox and Angielczyk [17] for autapomorphies of those clades, distinguishing them from basal taxa such as *Rastodon*). The current section is concerned primarily with distinguishing *Rastodon* from the phylogenetically volatile array of other, mostly generalized taxa at the base of Therochelonia.

Pylaecephalidae is a well-supported clade (containing the genera *Diictodon*, *Eosimops*, *Prosictodon*, and *Robertia*) of predominantly African dicynodonts with an unstable position in dicynodont phylogeny, having been recovered as either one of the most basal dicynodont groups [4,13,55,69–72] or as the sister-taxon of Emydopoidea within Therochelonia [24,49]. *Rastodon* lacks the key pylaecephalid synapomorphies of a precaniniform notch and bifurcate posterior margin of the dorsal process of the premaxilla. It also differs from all pylaecephalids in its proportionally longer temporal region, better-developed crests on the anterior rami of the pterygoids that converge into a longer, taller crista oesophagea, more elongate dorsal premaxillary process, dorsoventrally lower snout (even factoring in dorsoventral compression—in badly dorsoventrally crushed specimens of *Diictodon feliceps* [e.g., BP/1/494, SAM-PK-11563, USNM 23340] the snout remains tall, as it is the most robust part of the dicynodont cranium), and broader, lower occiput, with longer paroccipital processes that curve ventrolaterally, extending well below the ventral margins of the basal tubera (the short, stout paroccipital processes of pylaecephalids have a ventrolateral tip at the same level as the ventral margins of the basal tubera [57]). *Rastodon* can further be distinguished from *Diictodon* (but not the other pylaecephalid genera) by its broader median exposure of the parietals and larger postfrontal.

Eumantelliidae is currently monotypic (containing only *Pristerodon mackayi*), but this family requires revision; it is likely that ‘*P*. *mackayi*’ as currently conceived [73–74] contains multiple distinct species (C. Kammerer pers. obs.) This said, *Rastodon* can be distinguished from all specimens currently referred to *Pristerodon* by its proportionally longer snout and intertemporal region, intertemporal region that narrows posteriorly and lacks a significant postparietal contribution to the skull roof, more elongate dorsal process of the premaxilla (although some *Pristerodon* premaxillae have similar proportions, e.g., BP/1/3024), longer posterior processes of the frontals (extending past the mid-length of the pineal foramen; in all specimens of *Pristerodon* the posterior frontal processes terminate at the anterior margin of the pineal foramen at most), and proportionally broader occiput with longer, more ventrolaterally curved paroccipital processes. *Rastodon* can also be distinguished from *Pristerodon* by its preparietal morphology, making up the entire anterior half of the pineal boss and expanding anteriorly with a broadly rounded anterior edge. Although preparietal morphology is notoriously variable in anomodonts and generally not a useful species-specific character [4,75], all specimens of *Pristerodon* have a diamond-shaped preparietal, with narrow, pointed posterior and anterior tips. The sole exception may be NHMUK R4955 (holotype of *Palemydops platysoma*) which, uniquely among known dicynodont specimens, has a trident-shaped preparietal. *Palemydops platysoma* was considered a possible specimen of *Pristerodon mackayi* by Keyser [73], but in the end he deemed it a *nomen dubium* due to its incomplete preparation. More research on this specimen is required to confirm its possible relationship to *Pristerodon*, but it is clearly not the same as *Rastodon*.

Emydopoidea represents one of the three main therochelonian lineages and currently contains four families (Emydopidae, Kingoriidae, Myosauridae, and Cistecephalidae [59]). Cistecephalids have extremely short, broad skulls and are highly specialized for a burrowing lifestyle. The Early Triassic *Myosaurus*, the sole currently-recognized myosaurid, also has an unusually short skull, albeit with massive orbits compared to cistecephalids. Although somewhat more generalized in overall skull shape (with species of *Dicynodontoides* often historically confused with *Dicynodon* and *Oudenodon*; Kammerer et al. [4]), kingoriids are a highly autapomorphic group in their own right: their extremely narrow intertemporal regions (with the pineal foramen enveloped by the sagittal crest or absent) and complete (or nearly-complete) occlusion of the mandibular fenestra readily distinguish this group from *Rastodon*. Cistecephalids, myosaurids, and kingoriids are also distinguished from *Rastodon* by their shared absence of the postfrontal bone and expansion of the anterior orbital wall to close off the snout from the rest of the skull. Finally, *Rastodon* can be distinguished from *Emydops* (the sole recognized emydopid genus) by its longer, narrower intertemporal region (with a median trough for the parietals, unlike the flat-to-convex posterior skull roof in *Emydops*), generally lower skull and occiput, lack of an elongate, spike-like posterior protrustion on the paroccipital process, and well-developed pterygoid crests converging into the crista oesophagea (convergence of these crests is also absent in kingoriids). *Rastodon* can further be distinguished from all emydopoids (including the recently-redescribed *Digalodon*, of uncertain familial attribution; Kammerer et al. [49]) by the absence of an embayment of the palatal rim anterior to the caniniform process, keel-like extension of palatal rim posterior to the caniniform process, and jaw symphysis with shovel-shaped tip [59].

Within Bidentalia (the clade containing Cryptodontia, Dicynodontoidea, and all taxa more closely related to them than to emydopoids), very few generalized, basal forms are known; most bidentalian taxa are readily referable to either Cryptodontia or Dicynodontoidea. The only bidentalian taxa sometimes recovered outside of those groups are *Elph* and *Interpresosaurus* from the Late Permian of Russia [76] and *Katumbia* from the Late Permian of Tanzania and possibly Zambia [13,16]. These taxa are variously recovered (sometimes forming a clade, Elphidae) as either the basalmost bidentalians or at the base of Dicynodontoidea [4,24,49]. These taxa can be distinguished from *Rastodon* by their proportionally shorter, broader skulls, broader dorsal process of the premaxilla, caniniform processes at the same level as the anterior margin of the orbit, extensive overlap of the parietals by postorbitals in the intertemporal bar, and absence of an anterior process of the splenial (with the latter two characters being dicynodontoid synapomorphies).

Two basal (i.e., not oudenodontid, rhachiocephalid, or geikiid) genera are known in Cryptodontia: *Daqingshanodon* from the Late Permian of China and *Keyseria* from the Late Permian of South Africa [4]. *Rastodon* can be distinguished from *Daqingshanodon* by its proportionally longer skull (particularly the temporal and basicranial regions, but this might be related to the juvenile status of the holotype of *Daqingshanodon*), broader median exposure of the parietals, taller crista oesophagea, and absence of a sharp lateral ridge on the caniniform process. *Rastodon* additionally differs from *Daqingshanodon* (and oudenodontids, rhachiocephalids, and geikiids) in the absence of typical cryptodont synapomorphies: it has no postcaniniform crest and only a median, weakly-developed nasal boss. *Rastodon* also lacks a labial fossa (sensu Angielczyk and Kurkin [71]), which further distinguishes it from geikiids (and dicynodontoids).

In general appearance, the skull of *Rastodon* is most similar to that of *Keyseria*, a genus recently established by Kammerer et al. [4] for the species formerly known as ‘*Dicynodon*’ *benjamini*, known from two specimens from the Beaufort Group of South Africa. Kammerer et al. (2011:14) also described *Keyseria* as “remarkably generalized” and considered it a metataxon (i.e., lacking autapomorphies). Ventrally, the skulls of *Rastodon* and *Keyseria* exhibit a marked posterior angulation of the quadrate rami of the pterygoid and a similar anterodorsal slope of the basal tubera, forming ridges that converge towards the median pterygoid plate. However, *Rastodon* can be distinguished from *Keyseria* by its narrower intertemporal region (and consequently, less exposure of the parietals), absence of a well-developed ridge along the lateral premaxilla-maxilla suture, absence of a marked embayment anterior to the caniniform process, lower, anteroposteriorly broader caniniform process, better-developed ridges on the anterior rami of the pterygoid that converge into the crista oesophagea, relatively anterior position of the pineal foramen, and presence of a smaller pineal foramen with raised rim (in *Keyseria* the foramen is large and lacks any collar- or chimney-like boss).

### Phylogenetic position of *Rastodon* and implications for bidentalian origins

**Fig 7:**
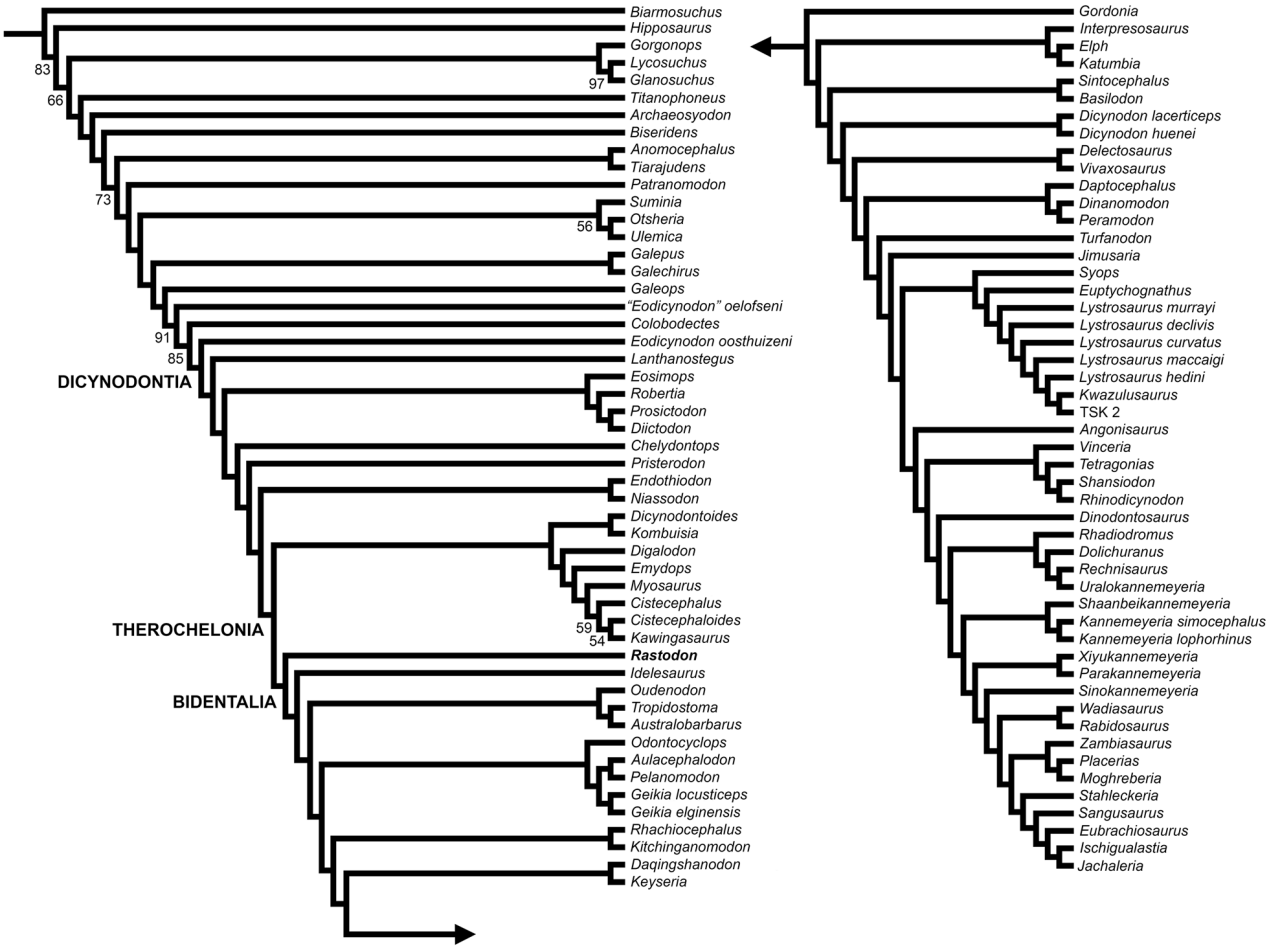
Phylogenetic position of *Rastodon procurvidens* within Dicynodontia based on the results of the phylogenetic analysis.

The phylogenetic analysis resulted in one most parsimonious tree with a tree length of 1018.763 steps. The consistency index (CI) is 0.238 and its retention index (RI) is of 0.712. *Rastodon* was found to nest within Bidentalia as the most basal member of this clade (Fig 7). The recovered tree topology is generally similar to that of other recent anomodont phylogenies (e.g. [15]), with some alterations. Beyond the addition of *Rastodon*, the current phylogeny differs from that of Kammerer et al. [49] in the extensive paraphyly of Cryptodontia, forming a grade at the base of Dicynodontoidea. It should be noted that this result has previously been recovered in certain iterations of the matrix [4] and character support for Cryptodontia is reliant primarily on continuous characters. *Niassodon* is recovered as the sister-taxon of *Endothiodon*, contra most earlier analyses ([15]; although see [77]) that recovered it as a kingoriid emydopoid, but consistent with its extensive postcanine dentition and *Endothiodon*-like palatines. Pylaecephalids are recovered outside of Therochelonia, in accordance with a variety of previous analyses [13,54,65,66,69]. Unusually, *Colobodectes* is recovered in a more basal position than *Eodicynodon oosthuizeni* (see [77] for this topology as well), suggesting instability at the base of Dicynodontia in this analysis.

The inclusion of *Rastodon* in Bidentalia is supported by the following characters: length of interpterygoid vacuity (character 8), ratio of mandibular fenestra (character 12) and absence of an expanded area in the midventral plate of the vomers (character 65). It is possible that this taxon could actually represent a basal cryptodont or dicynodontoid rather than a ‘stem’-bidentalian, but it is unlikely to represent a more basal taxon.

The major dicynodont subclade Bidentalia must have diverged by the middle Permian (based on the presence of the oudenodontid cryptodont *Australobarbarus* in the middle Permian Kotelnich fauna of Russia and on the ghost lineage implied by emydopoids of Tanzania), but members of this group are conspicuously absent in Guadalupian rocks of Africa. Despite intense study and a rich dicynodont fossil record, the *Eodicynodon* and *Tapinocephalus* AZs of South Africa have not produced any bidentalian fossils. The first record of a bidentalian in South Africa is the appearance of the oudenodontid cryptodont *Tropidostoma dubium* in its eponymous assemblage zone at the base of the Upper Permian. Given recent, detailed scrutiny of the *Tapinocephalus* AZ [78] it is unlikely that misidentification or inadequate sampling can explain the absence of bidentalians in the middle Permian record of South Africa. Rather, it is likely that the early evolution of this group was occurring outside of African basins. *Rastodon* represents the first example of a basal bidentalian occurring in the middle Permian (*Elph*, *Interpresosaurus*, *Katumbia*, *Keyseria and Daqingshanodon* are all late Permian taxa, and *Elph*, *Interpresosaurus* and *Katumbia* may represent dicynodontoids as noted above). Considering that true cryptodonts (as represented by *Australobarbarus*) were already present in Russia in the middle Permian, it seems that bidentalians radiated rapidly and achieved broad distribution early in their history. The reason for their exclusion from African basins is currently unknown. Further research into understudied middle Permian records (such as those of South America, Asia and African basins outside South Africa) is needed to better understand the origins of this important clade.

## Supporting Information

S1 Text Data matrix coded for *Rastodon procurvidens* based on Kammerer et al [49]. (NEX)
